# The prevalence and impact of workplace violence in community pharmacies: a mixed-methods study

**DOI:** 10.55730/1300-0144.6167

**Published:** 2025-12-01

**Authors:** Hilal İLBARS, Berna TERZİOĞLU BEBİTOĞLU, Seyhan HIDIROĞLU, Fatma Burcu DOĞANÇ, Yağız VAROL

**Affiliations:** 1 Faculty of Pharmacy, Başkent University, Ankara, Turkiye; 2 Department of Medical Pharmacology, Faculty of Medicine, Marmara University, İstanbul, Turkiye; 3 Department of Public Health, Faculty of Medicine, Marmara University, İstanbul, Turkiye; 4 Faculty of Medicine, Marmara University, İstanbul, Turkiye

**Keywords:** Workplace violence, work safety, community pharmacy, pharmacist, pharmacy technician, occupational health

## Abstract

**Background/aim:**

Pharmacists and pharmacy technicians employed in community pharmacies face a considerable risk of workplace violence, which can negatively influence their job satisfaction, productivity, and mental well-being. This study examined the prevalence and consequences of workplace violence in community pharmacies in Ankara through a mixed-methods design integrating cross-sectional survey data with qualitative insights.

**Materials and methods:**

The target population included pharmacists and pharmacy technicians. We examined demographic factors, psychological outcomes, and the nature of violent incidents. Data were obtained from a random sample of 355 community pharmacies using a 39-item survey and the depression anxiety stress scales (DASS-21), complemented by qualitative interviews with 12 pharmacists.

**Results:**

A total of 242 participants took part in the study: 174 pharmacists (71.9%; median age 46.0 years) and 68 pharmacy technicians (28.1%; median age 35.0 years). Overall, 63.2% reported at least one violent incident (58.0% of pharmacists; 76.5% of pharmacy technicians). Verbal violence emerged as the most prevalent form, with pharmacy technicians reporting significantly higher exposure than pharmacists. Perpetrators were most often patients or their relatives, while broader societal violence and deficiencies within the health system were frequently cited as contributing factors. Many participants refrained from taking legal action, attributing this to professional burnout and the perception of violence as an inherent aspect of their occupation. Findings from the DASS-21 revealed higher anxiety scores among pharmacy technicians compared to pharmacists. Among pharmacists, exposure to workplace violence was paradoxically associated with lower stress and depression scores, a pattern not observed among technicians.

**Conclusion:**

Workplace violence in community pharmacies is prevalent—particularly verbal abuse—and disproportionately impacts pharmacy technicians. Addressing this issue necessitates enhanced training, institutional support, and strengthened security measures, alongside extending white code legal protections to community pharmacies to improve safety and service quality.

## 1. Introduction

Workplace violence in the health care sector represents a major global public health issue that undermines the safety, psychological well-being, and job satisfaction of health professionals. International evidence shows that a substantial proportion of health care workers experience some form of violence annually; estimates indicate that 61.9% report exposure, with 42.5% subjected to nonphysical and 24.4% to physical violence. Among nonphysical forms, verbal abuse is the most prevalent, followed by threats and sexual harassment [1]. Studies conducted in Türkiye similarly report high prevalence rates: 44.7% of health care workers have experienced workplace violence [2], and 61.1% have reported verbal and/or physical assaults [3].

Workplace violence in community pharmacies has been documented worldwide, highlighting the urgent need for effective preventive and mitigation strategies. In Saudi Arabia, a 2025 systematic review reported prevalence ranging from 26% to 90.7% among physicians, pharmacists, and nurses [4]. In Australia, 91% of surveyed community pharmacists experienced or witnessed workplace violence within a 12-month period, including verbal abuse, bullying, and sexual harassment, leading to downstream consequences such as job turnover and insufficient postincident support [5]. Studies from the United Kingdom have likewise reported high levels of threats and assaults, with significant implications for the range of services pharmacists are willing to provide [6,7]. In Ireland, 77% of community pharmacies reported at least one violent event (verbal or physical) in the past year, with 18% reporting physical assaults and 63% expressing concern about workplace violence. The impact on employees was substantial, contributing to increased absenteeism and heightened fear among staff [8]. In Mansoura city, Egypt, 34.6% of pharmacy staff reported exposure to violence in the previous year, primarily verbal abuse [9]. In Nigeria, the prevalence of occupational violence among pharmacists reached 92.7%, with incidents frequently linked to long waiting times and patients’ dissatisfaction when their demands were not met [10]. Collectively, these findings underscore the global scope of the problem and emphasize the urgent need for comprehensive measures to safeguard pharmacy personnel.

Community pharmacies have also been recognized as potential access points for broader public health initiatives, including responses to domestic and sexual violence, owing to their accessibility and the high level of public trust they command [11,12]. To realize this potential, pharmacists must be equipped with adequate training, institutional support, and practical resources.

Given the widespread nature and serious consequences of workplace violence in community pharmacies, a multifaceted response is required, encompassing improved reporting mechanisms, postincident psychological support, and targeted professional training. This study aimed to determine the frequency of workplace violence experienced in community pharmacies in Ankara and to assess its psychological and professional impacts on those affected. By integrating quantitative and qualitative methods, this study sought to capture both the prevalence and the lived experiences of those encountering these challenges, and to develop data-informed recommendations for prevention and intervention.

## 2. Materials and methods

This mixed-methods study utilized a convergent parallel design, wherein quantitative and qualitative data were collected concurrently, analyzed independently, and integrated during interpretation to achieve a comprehensive understanding of workplace violence in community pharmacies. The study population comprised pharmacists and pharmacy technicians employed in community pharmacies in Ankara, Türkiye. According to 2024 data from the Turkish Medicines and Medical Devices Agency, Ankara has 2265 pharmacies. Assuming at least one pharmacist and one technician per pharmacy, the minimum sample size for the cross-sectional component was determined to be 355, based on a 95% confidence level and a 5% margin of error. Accordingly, 355 pharmacies were selected using a simple random sampling method from the Ankara pharmacy registry, and online surveys were distributed to pharmacists and pharmacy technicians working in these pharmacies. A web-based random number generator was employed to assign identification numbers to eligible pharmacies and ensure unbiased selection. In cases of nonresponse, the survey was resent once; if no response was obtained thereafter, the subsequent pharmacy on the registry was contacted. In total, 355 pharmacies were contacted, and 242 individuals agreed to participate, yielding a 68.2% overall response rate.

### 2.1. Data collection for the quantitative survey

Data were collected using a researcher-developed 39-item questionnaire grounded in the existing literature on workplace violence (Supplementary materials). Items were adapted from previously validated instruments assessing workplace violence and mental health outcomes, while additional items were developed de novo to capture context-specific experiences in community pharmacy settings. The questionnaire was pilot tested among pharmacy staff (n = 9) to ensure clarity, relevance, and comprehensibility, after which minor revisions were implemented. The short form of the depression anxiety stress scales (DASS-21), adapted for the Turkish population by Yılmaz et al., was used to assess depression, anxiety, and stress levels [13]. The original scale, developed by Lovibond et al., comprised 42 items, whereas the DASS-21 employs a 4-point Likert scale ranging from 0 to 3. Reported internal consistency values indicated Cronbach’s alpha coefficients of 0.67 for depression, 0.86 for anxiety, 0.67 for stress, and 0.87 for the overall scale [14]. DASS-21 subscale scores were computed by summing individual item responses within the depression, anxiety, and stress domains. Higher scores reflected greater symptom severity; results were categorized as normal, mild, moderate, severe, or extremely severe according to established cut-off points [13–14].

Categorical variables were summarized as frequencies and percentages, and chi-square tests were applied for group comparisons. Continuous variables were expressed as mean ± standard deviation, median, and range; normality was assessed using the Kolmogorov–Smirnov test. Student’s t-test was employed to compare means between two normally distributed groups, whereas the Mann–Whitney U test was applied for nonnormally distributed data. For comparisons involving more than two groups with nonnormal distributions, the Kruskal–Wallis H test was conducted. A significance level of p < 0.05 was considered statistically significant.

### 2.2. Data collection for the qualitative study

Qualitative data were collected through in-depth online interviews conducted with 12 community pharmacists in Ankara. A phenomenological research approach was adopted to explore participants’ lived experiences of workplace violence. Participants were recruited voluntarily using a snowball sampling technique. To maximize variation, pharmacists with prior experiences of workplace violence were purposively sampled, taking into account age, sex, pharmacy location, and years of professional experience. The first interview was conducted with a pharmacist known to have experienced workplace violence, followed by two pharmacists recommended by the initial participant. Recruitment continued until 10 interviews were completed, and two additional interviews were conducted to confirm data saturation. A semistructured interview guide (Supplementary materials) was used to explore the occurrence, perceived causes, and prevention strategies related to workplace violence. The mean interview duration was 23 min. Before each interview, participants were informed about the study objectives and provided written consent for audio recording. All personal data and audio recordings were kept strictly confidential and not disclosed to any third parties. Findings are reported exclusively in anonymized form.

Audio recordings were transcribed verbatim and subsequently converted into digital text format. Qualitative data were analyzed using thematic analysis based on Braun and Clarke’s seven-phase framework: (1) data familiarization, (2) generating initial codes, (3) searching for themes, (4) reviewing themes, (5) defining and naming themes, (6) producing the report, and (7) maintaining reflexivity and transparency throughout the process [15]. Analyses were conducted in ATLAS.ti version 24.0 (Scientific Software Development GmbH, Berlin, Germany). To enhance analytic rigor, five researchers independently participated in the coding process. Intercoder reliability was ensured through independent coding, followed by discussions until consensus was achieved, in accordance with Lincoln and Guba’s trustworthiness criteria [16]. Coding consistency was compared across researchers, and discrepancies were resolved through iterative consensus meetings. Final themes were collaboratively developed, and illustrative quotations were linked to participant characteristics (participant number, sex, years of experience, and pharmacy type) to enhance contextual depth and transferability. Direct quotations are presented to substantiate and illustrate the thematic findings.

## 3. Results

### 3.1. Cross-sectional findings

Of 242 participants, 71.9% (n = 174) were pharmacists and 28.1% (n = 68) were pharmacy technicians. The median age of pharmacists was 46.0 years (mean = 45.7; range: 24–68), while that of pharmacy technicians was 35.0 years (mean = 34.6; range: 23–49). Median years of professional experience were 22.0 for pharmacists (mean = 22.4; range: 1–42) and 14.0 for pharmacy technicians (mean = 13.9; range: 3–32). Participating pharmacies reported a median of 30 years at their current location (mean = 34.5; range: 1–50; n = 17). All participating pharmacies reported having some form of security measures in place.

Overall, 63.2% (n = 153) of participants reported experiencing at least one violent incident while working in a pharmacy—58.0% (n = 101) of pharmacists and 76.5% (n = 52) of pharmacy technicians. The most prevalent form of workplace violence was verbal abuse, followed by physical violence (Table 1). Technicians were significantly more likely than pharmacists to experience violence (p = 0.008; OR = 1.891, 95% CI: 1.152–3.103). Perpetrators were most frequently patients, followed by patients’ relatives and other customers (Table 1). No statistically significant differences were observed by timing of incidents (day versus night; weekdays versus weekend or public holidays; p > 0.05).

Contributing factors to workplace violence included the rise in overall societal violence—most frequently cited by participants—followed by systemic problems within the health care system (Table 2). Many participants reported responding verbally and initiating legal procedures; however, professional burnout and the perception that violence is “part of the job” were frequently cited as reasons for refraining from filing official complaints.

No statistically significant sex difference in exposure was observed among pharmacists (p = 0.784). Among pharmacy technicians, females reported significantly higher exposure to workplace violence than males (p < 0.001; OR = 4.205; 95% CI: 1.502–11.775). Among pharmacists, exposure was not associated with years of professional experience but demonstrated a weak positive correlation with age (p = 0.033; r = 0.16). Among pharmacy technicians, neither age nor years of experience showed a significant association with exposure (p = 0.275; p = 0.205).

Pharmacy district was not significantly associated with exposure to workplace violence (p > 0.05). Exposure significantly differed by pharmacy location type (p < 0.001); those working in pharmacies located within commercial areas reported more frequent incidents, whereas those in street-level settings reported the fewest (p < 0.001). On-call status, frequency of on-call shifts, number of pharmacists and technicians, and the presence of additional staff during on-call hours were not significantly associated with exposure (p > 0.05).

Prior training on workplace violence was uncommon and showed no significant association with exposure (p > 0.05). Participants who had previously experienced workplace violence reported significantly higher levels of moderate-to-high concern regarding workplace safety (pharmacists: p < 0.001; pharmacy technicians: p = 0.018).

### 3.2. DASS-21 findings

The outcomes of the DASS-21 assessment are presented in Table 3. Among pharmacists, depression was classified as normal in 75.3% (n = 131), mild in 18.4% (n = 32), and moderate in 6.3% (n = 11). Anxiety levels were normal in 86.2% (n = 150), mild in 10.9% (n = 19), and moderate in 2.9% (n = 5); all pharmacists scored within the normal range for stress. Among technicians, depression was normal in 58.8% (n = 40), mild in 23.5% (n = 16), and moderate in 17.6% (n = 12). Anxiety was normal in 75.0% (n = 51), mild in 13.2% (n = 9), moderate in 8.8% (n = 6), and severe in 2.9% (n = 2). For stress, all pharmacy technicians were within the normal range, except for one participant who was classified as mild. Pharmacy technicians had significantly higher anxiety scores than pharmacists (p = 0.042).

No statistically significant differences in DASS-21 scores were observed across sex, marital status, age group, education level, or job satisfaction (p > 0.05). Among pharmacists, voluntarily choosing the profession was not associated with DASS-21 scores; however, among pharmacy technicians, those who had chosen the profession willingly exhibited higher stress and anxiety scores (p = 0.010; p = 0.019).

Among pharmacists, exposure to workplace violence was unexpectedly associated with lower stress and depression scores (p = 0.013; p = 0.026, respectively). Among pharmacy technicians, exposure to workplace violence was not significantly associated with stress, depression, or anxiety (p > 0.05). When both groups were analyzed together, participants who reported experiencing sexual violence had significantly higher stress scores (p = 0.049). No statistically significant differences in DASS-21 scores were identified based on pharmacy district, type of location, on-call status, frequency of on-call shifts, presence of additional staff during on-call hours, or whether the pharmacy was located in a perceived safe area (p > 0.05).

### 3.3. Qualitative findings

A total of 12 community pharmacists participated in the qualitative phase of the study (four males and eight females), with a mean age of 35.5 years and an average of 13.6 years of professional experience. Participant characteristics are presented in Table 4. Thematic analysis revealed four overarching themes: (1) causes of violence, (2) reactions to violence, (3) emotional and professional impacts, and (4) protective measures and recommendations. The qualitative data reflected diverse experiences of workplace violence among pharmacists across various practice settings, with verbal abuse emerging as the predominant form. Several participants reported exposure to multiple forms of workplace violence, underscoring serious safety concerns within community pharmacy settings. The identified themes and subthemes captured multiple dimensions of these experiences, including contributing factors, emotional consequences, and strategies for prevention and coping.

#### 3.3.1. Themes and subthemes

Themes and subthemes are presented in Table 5.

##### 3.3.1.1. Causes of violence

Violence was primarily attributed to economic hardship, deficiencies in health policy (e.g., disputes over prescription regulations and medication copayments), unrealistic societal expectations, patient frustration, poor communication, and inadequate working conditions:

“Things like the pharmacist collecting the examination fee and medication copayments increase the financial burden on patients… economic reasons become one of the most important causes of violence.” (P1, F, 54, independent pharmacist, 31 years)

“I think there are two reasons: the public’s lack of sufficient knowledge—people don’t know where to find a solution—and a sense of helplessness and ignorance.” (P9, M, 27, independent pharmacist, 3 years)

“There seems to be a general problem in society… no one has any respect for anyone anymore, so the patient has no respect for the pharmacist either.” (P12, F, 36, independent pharmacist, 11 years)

“Since hospitals are often the focus of workplace violence discussions, pharmacists remain quite isolated in this context. They are not part of an institutional structure. Even when the ‘white code’ [emergency alert] system fails, hospital personnel still have security guards, nurses, or colleagues nearby. In community pharmacies, it’s different—you are completely alone.” (P1, F, 54, independent pharmacist, 31 years)

“They generally experience physical violence in more remote areas, farther from security measures. Such pharmacies are easier to access and therefore more vulnerable.” (P6, F, 38, independent pharmacist, 13 years)

##### 3.3.1.2. Reactions to violence

Pharmacists reported using panic buttons, adopting nonconfrontational deescalation strategies, and contacting law enforcement when necessary; however, many hesitation to formally report incidents due to fear of retaliation and limited trust in the legal system:

“Generally, our first preference, if it’s something we cannot resolve by communicating… is to call the police.” (P6, F, 38, independent pharmacist, 13 years)

“The police can’t really hold them; there’s a limit to how long they can be detained, and then it’s over. Even if they are jailed, there’s parole, or something happens, and they are released. Frankly, I don’t want to make these people a problem for myself. Of course, if I’m in real danger, calling the police and relying on them is the only option I have.” (P8, F, 55, independent pharmacist, 30 years)

“I don’t have much trust in the judicial process… It’s only about surviving the moment and avoiding physical harm.” (P10, M, 25, independent pharmacist, 1 year)

##### 3.3.1.3. Emotional and professional impact

Participants expressed emotions such as fear, anger, and disappointment. Some participants reported lasting impacts on trust and professional motivation, while others described resilience tempered by heightened vigilance. A few pharmacists reported considering leaving the profession altogether:

“The police can’t hold them either; there’s a limit to how long they can detain them, and then it’s over. Even if they are jailed, there’s parole, this and that, somehow... something happens, and they are released. Frankly, I don’t want to make these people a problem for myself. Of course, if I’m in real trouble, calling the police and relying on them is the only thing I can do.” (P8, F, 55, independent pharmacist, 30 years)

“I am afraid now. Beyond that, you feel sorry for your colleagues—when you empathize with them. I can say that my trust and affection for people in general are diminishing.” (P10, M, 25, independent pharmacist, 1 year)

“You experience a serious professional loss of motivation, and you start asking yourself, is it really worth struggling for this?” (P6, F, 38, independent pharmacist, 13 years)

“I have decided that I no longer want to be an independent pharmacy owner. I don’t want to deal with people in this way anymore. I don’t want to be face-to-face with patients so frequently or work in a role where I constantly provide direct patient counseling. I can say that I have become genuinely disillusioned with the profession because of this.” (P9, M, 27, independent pharmacist, 3 years)

##### 3.3.1.4. Protective measures and recommendations

Participants recommended improving physical security measures (e.g., cameras, shutters, and mobile alert systems), enhancing institutional support, strengthening legal regulations, extending the white code protections to community pharmacies, and increasing public education efforts:

“Penalties could be increased. Beyond that, education is essential. It’s not something that will change immediately, but perhaps through films and TV series—rather than showing mafia-type characters—the respectability of doctors and pharmacists could be emphasized more.” (P8, F, 55, independent pharmacist, 30 years)

“The white code system should perhaps be extended to include incidents of violence in pharmacies. The process of filing an official statement and initiating a public lawsuit could serve as a deterrent.” (P12, F, 36, independent pharmacist, 11 years)

“We are positioned as first-class healthcare professionals—legally we are. The ministry uses this definition when it suits them, but the issue of professional discreditation needs to be addressed. Moreover, they still fail to protect physicians in hospitals. The discourse on social media—such as ‘the pharmacist is black-marketing medicines’ or ‘they have a deal with the company,’—must be eliminated. I expect that this aggressive public energy should not be redirected toward pharmacists themselves.” (P6, F, 38, independent pharmacist, 13 years)

“I believe patients should be properly informed about the workflow and procedures in the pharmacy. There should be points of communication where they understand that the person in front of them cannot always provide a solution. Patients often assume that we can solve everything but choose not to, thinking we want to make things difficult for them. This misunderstanding, arising from a lack of knowledge, can easily trigger anger.” (P9, M, 27, independent pharmacist, 3 years)

## 4. Discussion

Workplace violence in community pharmacies represents a pervasive occupational hazard that compromises safety, diminishes job satisfaction, and jeopardizes the quality of pharmaceutical care delivery. This study is among the first mixed-methods investigations examining the prevalence, nature, and consequences of workplace violence in community pharmacies in Ankara, with particular attention to differences between pharmacists and pharmacy technicians.

Quantitatively, more than half of pharmacists (58.0%) and over three-quarters of pharmacy technicians (76.5%) reported experiencing workplace violence—predominantly verbal abuse (92.1% and 94.2%, respectively)—followed by threats, bullying, and, less frequently, physical assault and sexual harassment. Pharmacy technicians faced a significantly higher risk of exposure. Qualitative findings help elucidate this disparity: technicians perform highly frontline, patient-facing tasks (e.g., processing payments, managing logistics), often becoming the first recipients of patient frustration. Systemic pressures—including economic instability, out-of-pocket expenses, and stringent prescription regulations—further exacerbate tensions in pharmacy practice. Compared with findings from southeast Europe, variations in role delineation, legal protections, and societal perceptions of professional authority may contribute to differences in risk profiles [17].

Sex-based patterns were also evident: among pharmacy technicians, female participants reported significantly higher exposure to workplace violence, whereas no statistically significant sex differences were identified among pharmacists. This finding underscores the intersection between professional role and sex in shaping vulnerability to workplace aggression.

The integrated analysis suggests that workplace violence has become a normalized aspect of daily community pharmacy practice. While quantitative findings delineate prevalence, qualitative narratives elucidate underlying mechanisms—such as dissatisfaction with medication prices, prescription restrictions, and waiting times that often escalate into confrontations at the counter. Interventions should integrate deescalation training, institutional support mechanisms, clearer role delineation, and stronger legal protections—such as extending white code provisions to community pharmacies.

A recent study examining the risk of workplace violence among pharmacists and pharmacy technicians in community pharmacies across southeast Europe reported that more than 80% of participants had experienced verbal violence, while over 20% had been exposed to physical or sexual violence [17]. That study found no statistically significant differences between pharmacists and pharmacy technicians, nor across sex, age groups, or national contexts. In contrast, our findings revealed a clear disparity: pharmacy technicians were significantly more likely than pharmacists to report experiencing workplace violence (76.5% versus 58.0%). Despite these differences, both studies converge on several key points: verbal abuse remains by far the most prevalent form of violence, and patients or clients are consistently identified as the primary perpetrators. Moreover, our qualitative findings corroborate this pattern, as participants frequently described patient frustration related to economic hardship, prescription regulations, and long waiting times as triggers for aggression. Taken together, these findings highlight that although prevalence rates and role-specific risks may vary across contexts, community pharmacies consistently constitute vulnerable healthcare settings, underscoring the need for targeted preventive interventions at both local and international levels.

A recent metaanalysis estimated a pooled prevalence of 45% among pharmacists, with 39% reporting violent incidents within the preceding 12 months; verbal abuse (50%), threats (42%), and physical assaults (27%) were identified as the most common forms of workplace violence [18]. Our findings align with this global evidence, as verbal abuse emerged as the predominant form of workplace violence, reported by 92.1% of pharmacists and 94.2% of pharmacy technicians. However, the overall prevalence observed in our study was even higher, with 58.0% of pharmacists and 76.5% of pharmacy technicians reporting exposure to workplace violence. These elevated rates—particularly among pharmacy technicians—suggest that community pharmacies in Türkiye may face a greater burden than international averages, emphasizing the urgent need for systemic protective measures, such as expanding the white code program to community pharmacies, along with workplace-level interventions including deescalation training and structured staff support mechanisms.

Workplace violence in community pharmacies exerts a substantial impact on the mental health of pharmacists, contributing to increased stress, anxiety, burnout, and symptoms consistent with posttraumatic stress disorder [8,9]. In our study, concerns regarding workplace violence were particularly pronounced among individuals with previous exposure, showing a statistically significant correlation between prior incidents and heightened perceptions of safety risk. Accordingly, anxiety scores on the depression anxiety stress scales (DASS-21) were significantly higher among participants exposed to workplace violence, with pharmacy technicians exhibiting greater anxiety levels than pharmacists. Furthermore, participants who had experienced sexual violence (two pharmacists and one technician) demonstrated significantly elevated stress scores.

Pharmacy technicians exhibited significantly higher anxiety scores compared with pharmacists. Rates of mild and moderate depressive symptoms were also higher among pharmacy technicians. This disparity may reflect technicians’ higher exposure to violence, differences in professional status, or variation in coping mechanisms for job-related stress. Interestingly, among pharmacists, those with prior exposure to workplace violence displayed lower stress and depression scores, suggesting the potential development of resilience or adaptive coping strategies over time. In contrast, no statistically significant association was observed between exposure to workplace violence and mental health outcomes among pharmacy technicians.

Quantitative DASS-21 findings therefore suggested a notable degree of resilience among pharmacists, with most scores within the normal range and even lower stress and depression levels among those exposed to workplace violence. However, qualitative findings revealed the underlying complexity behind this apparent resilience. Pharmacists described profound emotional consequences—including fear, anger, and disappointment—that the quantitative scales did not fully capture. Their perceived resilience appears to reflect professional adaptation rather than the absence of psychological impact; they continue to work with heightened vigilance despite underlying emotional distress. For some pharmacists, this emotional burden becomes overwhelming, prompting contemplation of leaving the profession—an outcome often overlooked by quantitative measures alone.

Qualitative data further add critical depth to understanding how pharmacists respond to workplace violence. Survey findings indicated that pharmacists most commonly responded verbally or pursued legal action, yet many were reluctant to formally report incidents due to burnout and emotional fatigue. Interview narratives elucidated this reluctance, citing fear of retaliation and profound mistrust in a legal system perceived as slow and ineffective. This dynamic helps explain the observed discrepancy between experiencing workplace violence and formally reporting such incidents. Additionally, qualitative accounts highlighted proactive protective strategies—such as activating panic buttons and employing deescalation techniques—that illustrate how pharmacists manage daily occupational threats in practice.

In a cross-sectional study assessing burnout levels and prevalence among community pharmacists in Ankara, the Maslach Burnout Inventory was administered to 251 pharmacists [19]. The mean emotional exhaustion score was 16.84 (SD = 6.25), the mean depersonalization score was 4 (range: 0–14), and the mean personal accomplishment score was 22 (range: 9–32). Age, marital status, professional experience, job satisfaction, workload, time pressure, perceived stress, and satisfaction with customers were all significantly associated with burnout levels among pharmacists [19].

In Lebanon, the overall prevalence of burnout among pharmacists was reported at 43.5%, primarily driven by high emotional exhaustion (36.9%) [20]. Factors associated with higher burnout levels included older age, holding a Bachelor of Science in Pharmacy degree, participation in student training, lack of involvement in procurement, divided attention at work, overall career dissatisfaction, and dissatisfaction or neutrality regarding work–life balance. Low personal accomplishment was reported by 49.5% of participants [20].

Our findings reveal a substantial psychological burden within community pharmacy practice, consistent with previous burnout research, and emphasize the urgent need for systemic interventions to safeguard the mental well-being of pharmacy personnel.

An important contributing factor is the increasing workload experienced by pharmacists. Long hours, understaffing, and limited support were identified as key stressors contributing to workplace conflicts and violence. A UK review reported that pharmacists’ workload has increased, with most time still devoted to dispensing activities; while heavy workload is associated with reduced well-being, strong evidence of a direct patient safety risk remains limited [21].

Violent incidents also contribute to job mobility, with many pharmacists transferring to other community pharmacy settings [5]. In our study, exposure to workplace violence diminished professional motivation, yet many pharmacists remained committed to their profession despite these challenges.

Workplace violence is strongly associated with absenteeism and heightened fear among pharmacy employees [8]. Participants also reported feelings of anger and disappointment, which further undermine professional identity and interpersonal trust.

Underreporting of incidents and lack of institutional support were common among participants. In our study, pharmacists expressed distrust in the judicial system and concern over ineffective legal outcomes, which discouraged them from seeking justice. In another study, more than 90% of pharmacy staff reported receiving no employer or external assistance following robbery incidents [17]. Only 0.6% of our participants had ever received formal workplace violence training [12]. Pharmacists implemented self-initiated safety measures—such as rotating staff, using safety applications, and installing alarm systems or controlled-access doors—and recommended stricter legal penalties, enhanced public education, and a stronger police presence.

Contributing factors included long waiting times, refusal to meet aggressors’ demands, poor communication [10], and broader economic and political tensions, unmet patient expectations, and challenging working conditions. Financial instability, disputes over copayments, and controlled substances often triggered conflict. Long working hours, chronic understaffing, and inadequate security measures further exacerbated the risk of workplace violence.

A workforce retention study conducted in Ireland reported similar concerns, including heavy workloads, poor working conditions, excessive regulatory burden, and negative health impacts [22]. The study’s recommendations—improved employment conditions, greater recognition of professional roles, career advancement opportunities, and regulatory reform—closely align with the views expressed by our participants.

A study conducted in Mansoura City identified working night shifts and younger age (19–40 years) as significant predictors of experiencing workplace violence among pharmacists and pharmacy assistants [9].

In our study, no statistically significant differences were observed by age or the timing of violent incidents (day versus night, weekdays versus weekends or public holidays). Instead, professional role emerged as the strongest predictor, with pharmacy technicians experiencing substantially higher levels of workplace violence than pharmacists. This finding highlights how contextual and occupational factors collectively shape vulnerability to workplace violence across different pharmacy settings.

The prevalence of workplace violence influences the range of services pharmacists are willing to provide, with safety concerns directly impacting professional practice and patient interaction [5,7]. Participants emphasized the urgent need for enhanced security measures and greater organizational support to mitigate these impacts.

Participants also underscored the absence of the white code system for community pharmacists. Extending this system could ensure formal documentation of incidents, facilitate a more systematic legal response, and enhance workplace safety while deterring future acts of violence.

The qualitative recommendations offer a clear path forward by directly addressing the underlying causes and multifaceted impacts identified across both quantitative and qualitative datasets. Participants’ calls for improved physical security (e.g., cameras, shutters), stronger institutional support, stricter legal sanctions, enhanced public education, and greater societal recognition directly respond to the systemic shortcomings and safety gaps identified in this study.

### 4.1. Strengths and limitations

This study provides critical insights as the first mixed-methods investigation of workplace violence among community pharmacists in Türkiye’s capital city. It identifies key contributing factors, explores the emotional impact—particularly the high prevalence of verbal abuse directed toward pharmacy technicians—and underscores the need for structured mental health support, workforce retention strategies, and clearer workplace violence management frameworks.

The study’s limitations include reliance on self-reported data, which may be affected by recall or social desirability bias, and the use of a single-city sample, which limits generalizability. The qualitative component employed snowball sampling, which may have introduced selection bias, as initial participants were recruited from individuals known to have experienced workplace violence. Although efforts were made to include diverse sexes, ages, and pharmacy types, the findings may not fully represent all community pharmacy staff. Addressing these limitations in future research could enhance understanding and guide the development of more comprehensive, evidence-based strategies to strengthen pharmacy safety and promote staff well-being.

## 5. Conclusion

Workplace violence in community pharmacies is widespread, with verbal abuse emerging as the predominant form. Its consequences include psychological distress, professional demotivation, and workforce instability, compounded by persistent underreporting and limited institutional support. Contributing factors encompass long waiting times, unmet patient expectations, and communication breakdowns. A comprehensive, multilevel response—integrating staff training, organizational support mechanisms, strengthened security infrastructure, and coordinated policy action—is essential to protect pharmacy personnel and ensure the sustainability and quality of pharmaceutical care services.

## Supplementary Information





## Figures and Tables

**Table 1:** Characteristics of workplace violence experienced in community pharmacies.

Characteristics of violence	Pharmacists (%)	Pharmacy technicians (%)
**Type of violence**		
Physical violence	4.0%	1.9%
Verbal violence	59.2%	77.9%
Sexual violence	1.1%	1.9%
Theft/robbery	1.7%	1 (1.9%)
**Perpetrator of violence**		
Patient	65.3%	48.1%
Patient’s relative	29.7%	42.3%
Customer	5.0%	9.6%
Timing of violence incidents* (Multiple answers)		
During daytime working hours	60.3%	76.5%
During night shifts	96.0%	95.6%
On weekends and holidays	63.8%	88.2%

**Table 2:** Common contributing factors to workplace violence in community pharmacies.

Characteristics	Pharmacists n (%)	Pharmacy technicians n (%)
Easy accessibility of pharmacies	139 (79.9%)	61 (89.7%)
Rising violence in society	166 (95.4%)	66 (97.1%)
Substance abuse among perpetrators	119 (68.4%)	61 (89.7%)
Mental health issues among perpetrators	79 (45.4%)	34 (50.0%)
Issues in the healthcare system	162 (93.1%)	67 (98.5%)
Economic instability	151 (86.8%)	66 (97.1%)
Lack of consultation services	33 (19.0%)	27 (39.7%)

**Table 3:** DASS-21 scores of study participants.

	Pharmacists		Pharmacy technicians		p-value	r
	Median	Min–max	Median	Min–max		
Depression	3.0	0–8	3.0	0–8	0.415	0.052
Anxiety	2.0	0–7	2.0	0–7	0.042	0.131
Stress	4.0	0–7	4.5	0–7	0.059	0.121

**Table 4:** Demographic and professional characteristics of qualitative study participants.

Participant No	Age	Sex	Years of work	Pharmacy location	Experience of violence
P1	54	Female	31	On the street	Verbal, physical, sexual, robbery/theft
P2	36	Male	13	On the street	Verbal
P3	29	Female	3	On the street	None
P4	43	Female	17	Across from a hospital	Verbal
P5	36	Female	9	On the street	Verbal
P6	38	Female	13	On the street	Verbal
P7	56	Female	31	Neighborhood pharmacy	Verbal, robbery/theft
P8	55	Female	30	Across from a hospital	Verbal, physical
P9	27	Male	3	Across from a health center	Verbal
P10	25	Male	1	On the street	None
P11	26	Male	2	Neighborhood pharmacy	Verbal
P12	36	Female	11	On the street	Verbal

**Table 5:** Themes and sub-themes

Themes	Sub-themes
1. Causes of violence	1.1. Economic and Policy-Related Issues 1.2. Patient and Society-Related Circumstances 1.3. Communication and Relationship-Related Problems 1.4. Issues Related to Pharmacists’ Working Conditions
2. Reactions to violence	2.1. Reactions Following Violence 2.2. Problems Related to the Judiciary After Violence
3. Emotional and professional impact	3.1. Post-Violence Emotions 3.2. The Effects of Violence on the Profession
4. Protective measures and recommendations	4.1. Existing Security Measures in the Pharmacy 4.2. Additional Measures Taken After a Violent Incident 4.3. Measures That Need to Be Taken Against Violence
