# Supplementary material for: The prevalence and impact of workplace violence in community pharmacies: a mixed-methods study
Source: Turk J Med Sci. 2025 Dec 1;56(1):333–43. doi: 10.55730/1300-0144.6167 (PMC12974294; doi:10.55730/1300-0144.6167)
Supplement: Supplementary file 1 [file Appendix_1_39_item_survey_EN.docx]

# Appendix 1.

# 39-item Survey Form

**1)** Your gender:
□ Female  □ Male  □ Prefer not to say

**2)** Year of birth: ………

**3)** Marital status:
□ Married □ Single □ Divorced □ Widowed

**4)** Educational background:

□ Primary school
□ Secondary school
□ High school or equivalent
□ Vocational school / Associate degree
□ University / Bachelor’s degree
□ Master’s degree
□ Doctorate

**5)** Your profession:
□ Pharmacist
□ Pharmacy technician
□ Auxiliary staff
□ Other

**6)** How many years have you been practicing this profession? Please specify: ………

**7)** District where your pharmacy is located: ………

**8)** How many years has your pharmacy been operating in the same location? Please specify: ………

**9)** What type of area is your pharmacy located in?

□ Across from a hospital
□ Across from a primary care center (Family Health Center)
□ Neighborhood pharmacy
□ Shopping mall pharmacy
□ Other (please specify): ………

**10)** Did you choose your profession willingly?
□ Yes □ No

**11)** Are you satisfied with your profession?
□ Satisfied □ Partially □ Not satisfied

**12)** Is there another pharmacist working in your pharmacy?
□ Yes □ No

**13)** If yes, how many pharmacists (other than you) are employed in your pharmacy? Please specify: ………

**14)** How many pharmacy technicians are employed in your pharmacy (other than you)?
□ 1 □ 2 □ 3 □ 4 □ 5 or more

**15)** How many auxiliary staff are employed in your pharmacy (other than you)?
□ 1 □ 2 □ 3 □ 4 □ 5 or more

**16)** Do you work night shifts (on-call duty)?
□ Yes □ No

**17)** If yes, how often do you work night shifts?

□ Once a month

□ Twice a month

□ Three times a month
□ Once every two months

□ Other (please specify): ………

**18)** Do you employ additional staff during night shifts?
□ Yes □ No

**19)** If yes, how many additional staff do you employ?
□ 1 □ 2 □ 3 or more

**20)** During your time working in the pharmacy, have you ever been exposed to violent behavior?
□ Yes □ No

**21)** If your answer is yes, what type of incident did you experience?
□ Physical violence (e.g., punching, kicking, slapping, stabbing, shooting, biting, physical contact, etc.)
□ Verbal violence (e.g., threats, insults, swearing, humiliation)
□ Sexual violence (any unwanted, unsolicited, and unwelcome sexually aggressive behavior that causes distress, intimidation, humiliation, or shame)
□ Robbery (theft with force or threats)

**22)** Who committed the act of violence?
□ Patient □ Patient’s relative □ Customer □ Other (please specify): ………

**23)** What was the gender of the perpetrator?
□ Male □ Female

**24)** During your time in the pharmacy, has any of your auxiliary staff been exposed to violent behavior?
□ Yes □ No

**25)** If yes, what type of violent incident occurred?
□ Physical violence
□ Verbal violence
□ Sexual violence

**26)** Please indicate the frequency with which you personally experienced the following types of violence:
- Physical violence: ( ) Once ( ) 2–4 times ( ) 4–10 times ( ) More than 10 times
- Verbal violence: ( ) Once ( ) 2–4 times ( ) 4–10 times ( ) More than 10 times
- Sexual violence: ( ) Once ( ) 2–4 times ( ) 4–10 times ( ) More than 10 times

**27)** Do you think your pharmacy is located in a safe area?
□ Yes □ No

**28)** If yes, what kind of security measures have you taken? (Multiple choices allowed)

1. Alarm
2. Camera
3. Additional locks
4. Extra lighting
5. Shutter protection
6. Licensed firearm

7. No security measures

**29)** In your opinion, at what time of day do violent incidents mostly occur?
1. During daytime working hours
2. At night while on call duty
3. On weekends and holidays

**30)** If you were exposed to violence (verbal, physical, sexual, robbery), how did you respond? (Mark one option only)
1. I did not react
2. I tried to defend myself physically
3. I responded verbally
4. I called the police

**31)** What legal actions did you take after experiencing violence? (Multiple choices allowed)
1. Reported to the professional chamber
2. Filed a complaint to judicial authorities
3. Reported to law enforcement
4. Did not report to any authority

**32)** After filing a judicial complaint, did you follow up on the process regarding the person(s) you complained about?
□ Yes □ No

**33)** What was your reason for not filing a judicial complaint? (Mark one option only)

1. The judicial process is too lengthy
2. I considered it part of my profession
3. Fear of harm due to threats
4. Professional burnout/fatigue

**34)** In your opinion, what are the reasons for the increase in violent incidents against pharmacies? (Multiple choices allowed)
1. Easy accessibility of pharmacies
2. Increase in violence in society in general
3. Psychiatric illness of the perpetrator (e.g., schizophrenia, depression)
4. Substance abuse by the perpetrator
5. Problems related to the healthcare system (e.g., co-payment of medicines, unavailability of drugs in the market, issues with reimbursement systems)
6. Current unstable economic conditions in society
7. Problems related to consultancy services
8. Other (please specify): ………

**35)** What type of safety strategies would make you feel more secure against violence in pharmacies? (Multiple choices allowed)

1. Security personnel
2. Metal detector
3. Higher counters
4. Training (e.g., strategies to prevent workplace violence, communication skills, conflict resolution, self-defense, etc.)

5. Increased police/gendarmerie patrols during night shifts
6. Panic button
7. Bulletproof glass
8. Storing critical medicines in a coded safe
9. Applying the “white code” system (used in hospitals) also to community pharmacies

**36)** Have you ever participated in any training program (congress, panel, etc.) related to workplace violence?
□ Yes □ No

**37)** Do you feel concerned about work-related violence?
□ None □ Low □ Moderate □ High

**38)** How frequently are you exposed to verbal violence?

□ Every day
□ Once a week
□ More than once a week
□ Once a month
□ More than once a month
□ Once or twice a year

**39)** How frequently are you exposed to physical violence?

□ Every day
□ Once a week
□ More than once a week
□ Once a month
□ More than once a month
□ Once or twice a year
