# Supplementary material for: The prevalence and impact of workplace violence in community pharmacies: a mixed-methods study
Source: Turk J Med Sci. 2025 Dec 1;56(1):333–43. doi: 10.55730/1300-0144.6167 (PMC12974294; doi:10.55730/1300-0144.6167)
Supplement: Supplementary file 2 [file Appendix_2_Semi_Structured_Interview_EN.docx]

# Appendix 2.

# Semi-Structured Interview Form on Violence in Pharmacies

**1)** How many years have you been working as a community pharmacist? Where is your pharmacy located? (e.g., on a main street, in a shopping mall, in a neighborhood, etc.)

**2)** Have you ever experienced violence while working in the pharmacy? Could you describe how the incident occurred? In your opinion, what was the reason for the violence? *(Physical / Verbal / Sexual / Robbery should be asked separately.) (If the participant has not personally experienced it, they may be asked if they know of another pharmacist who has. It can also be asked whether they have heard about such incidents in the news. If they say they have never heard of it, general questions about violence in healthcare can be asked.)*

**3)** What did you do after experiencing violence? (*Did you react? For example: shouting, verbal response, hitting, reporting to the police. Were the police called to the scene? Did you give a statement afterwards?)* Did a legal process begin? Did you follow up on the legal process? How did it conclude? If you did not report to law enforcement, why not? Do you trust the judicial system in Türkiye?

**4)** What additional security measures did you take in your pharmacy after experiencing violence?

**5)** How did you feel after experiencing violence? How did the violent incident affect your job satisfaction? After experiencing violence, did you engage in less dialogue with people?

**6)** In your opinion, what measures should be taken in Türkiye to prevent violence in pharmacies?
